# 249 TP53 mutation has high prevalence and is correlated with larger and poorly differentiated HCC in Brazilian patients

**DOI:** 10.1186/1471-2407-9-204

**Published:** 2009-06-26

**Authors:** Jeronimo A Nogueira, Suzane K Ono-Nita, Marcelo E Nita, Marcelo MT de Souza, Eliane P do Carmo, Evandro S Mello, Cristovan Scapulatempo, Denise C Paranaguá-Vezozzo, Flair J Carrilho, Venancio AF Alves

**Affiliations:** 1 Department of Gastroenterology, University of São Paulo School of Medicine, São Paulo, SP, Brazil; 2 Department of Pathology, University of São Paulo School of Medicine, São Paulo, Brazil

## Abstract

**Background:**

Ser-249 TP53 mutation (249^Ser^) is a molecular evidence for aflatoxin-related carcinogenesis in Hepatocellular Carcinoma (HCC) and it is frequent in some African and Asian regions, but it is unusual in Western countries. HBV has been claimed to add a synergic effect on genesis of this particular mutation with aflatoxin. The aim of this study was to investigate the frequency of 249^Ser ^mutation in HCC from patients in Brazil.

**Methods:**

We studied 74 HCC formalin fixed paraffin blocks samples of patients whom underwent surgical resection in Brazil. 249^Ser ^mutation was analyzed by RFLP and DNA sequencing. HBV DNA presence was determined by Real-Time PCR.

**Results:**

249^Ser ^mutation was found in 21/74 (28%) samples while HBV DNA was detected in 13/74 (16%). 249^Ser ^mutation was detected in 21/74 samples by RFLP assay, of which 14 were confirmed by 249^Ser ^mutant-specific PCR, and 12 by nucleic acid sequencing. All HCC cases with p53-249ser mutation displayed also wild-type p53 sequences. Poorly differentiated HCC was more likely to have 249^Ser ^mutation (OR = 2.415, 95% CI = 1.001 – 5.824, p = 0.05). The mean size of 249^Ser ^HCC tumor was 9.4 cm versus 5.5 cm on wild type HCC (p = 0.012). HBV DNA detection was not related to 249^Ser ^mutation.

**Conclusion:**

Our results indicate that 249^Ser ^mutation is a HCC important factor of carcinogenesis in Brazil and it is associated to large and poorly differentiated tumors.

## Background

Many factors may lead to pre-malignant conditions related to the development of hepatocellular carcinoma (HCC), including Hepatitis B and C virus infection (HBV, HCV), alcohol intake and ingestion of food product with high concentration of mycotoxins such as Aflatoxin B1 (AFB1), which can be found in some developing countries [1]. Several of these factors have been shown capable of altering the expression of genes responsible for cell growth regulation [2,3].

It has been widely acknowledged that HCC development is strongly related to environment and socio-economic factors [4], leading to major differences not only in the incidence but also in molecular pathways of liver carcinogenesis in different geographic regions. Accordingly, mutations at TP53 gene are frequent in HCC patients from Africa and China, where this kind of tumor is highly incident, in sharp contrast to what has been reported from Europe and North America [5]. Further evidences for a direct carcinogenic effect of HBV and AFB1 is the finding which in countries where high rates of both ones are present and, furthermore, HBV infection is contracted in the early years of life, a higher frequency of HCC in non-cirrhotic liver is observed. On the other hand, in developed countries, HCC seems to be more related to HCV infection and to ethanol intake[6,7,5].

Rather than occurring at random, mutations along TP53 in HCC happen in "hot-spots", the most common of them is at codon 249 in exon 7, responsible for almost 40% of TP53 mutations reported in this neoplasm (IARC, 2004). It is referred as 249^Ser^, because of a conversion of G (guanine) into T (thymine) resulting in Arginine → Serine mutation in p53 protein. This event was first reported in 1991 simultaneously by two different research groups [8,9], independently demonstrating a strong relation of this mutation to a dietary exposure of AFB1. Latter it was described the geographical distribution of this specific mutation and its relation to AFB1 exposure [10].

Evidence from several other laboratories has further confirmed the strict relation between AFB1 and 249^Ser ^mutation of TP53: Jackson and co-workers [11] found this mutation in the serum of 46.7% HCC patients in Qidong (China). In a similar study from Gambia[12], this mutation was found in 36% of HCC patients sera, in 15% cirrhotic patients and in 6% of the control group.

Although the pathway on which aflatoxin induces this specific mutation, it is not totally elucidated, it is known that AFB1 itself is not the carcinogenic substance, but its second metabolite [13]. After being ingested along with the food, AFB1 is metabolized by CYP450 complex enzymes resulting on formation of AFB1-exo-8,9-epoxide. This specific metabolite has the capacity to make a covalent binding to DNA nucleotides leading adducts, the main of which resulting from the interaction between the epoxide and guanine: AFB1-N7-Gua [14]. It has been demonstrated that AFB1 can interact with 20% of the bases between exon 5 and exon 8 of TP53 – 85% of them were guanines [15]. Besides this hot-spot, Denissenko [16] detected adducts formation in other codons of exon 7 and 8 of TP53.

Studies indicate that the main risk for the adducts formation is the incapacity of metabolism phase 2 enzymes, especially isoforms of Glutatione S-Transferase on clearing AFB1-exo-8,9-epoxide [17].

The carcinogenic process derived from HBV infection has been associated to the expression of HBx oncoprotein [18]. Kim et al [19] in 1991, had already shown the role of HBx in neoplastic transformation in transgenic mice and the expression of this viral oncoprotein has been observed in more than half of HBV related HCC [20]. Relationship between HBV infection and p53 function is still controversial. Hosono and co-workers [21] did not find any association. Recently it was concluded that HBV transfection may lead to an abnormal expression of p53 in cell culture [22]. Studies with patient exposed to AFB1 and infected with HBV suggest a toxin-virus interaction where HBV is responsible to selectivity of 249^Ser ^mutation [23].

The aim of this study was to assess the frequency of TP53 249^Ser ^mutation in HCC samples from patients in Brazil as well as its eventual relation to the presence of HBV DNA in hepatocytes by Real-Time PCR.

## Methods

### Patients

Formalin fixed paraffin embedded (FFPE) blocks diagnosed as HCC from 80 patients from Division of Surgical Pathology at the Hospital das Clínicas of University of São Paulo School of Medicine (51 samples) and from Oswaldo Cruz German Hospital (29 samples) were selected from patients who underwent liver resection or liver transplantation from 1998 to 2005. Six patients were excluded because DNA was not amplified by PCR, therefore analysis included 74 patients. The characteristics of patients are described in table 1.

**Table 1:** Patients Characteristics

Characteristic	Number	(%)
Gender		
Male	53	71.6
Female	21	28.4
Age, years	54.04 ± 17,3^1^	7 – 80^2^
Viral etiology		
HBV	13	16
HCV	27	48.2^3^
HBV + HCV	3	5.4^3^
No virological data	12	21.4
Cirrhosis	49	66.2
Tumor size	6.58 + 5.9^1^	0.5 – 24^2^
Tumor differentiation grade		
G1	1	1.4
G2	30	40.5
G3	34	45.9
G4	9	12.2
Geographical area		
North	1	2.3
Northeast	6	13.9
Southeast	35	81.3
Outside Brazil	1	2.3

^1^Mean ± SD; ^2^Range; ^3 ^Sample size: 56

Additionally, 17 FFPE blocks of normal livers from patients who died from unrelated diseases necropsied at the Department of Pathology of University of São Paulo School of Medicine served as control group.

This study was approved by the Investigational Review Board of the University of Sao Paulo School of Medicine.

### DNA extraction

In order to avoid inter sample contamination, it was used a different sterile blade for each paraffin block. Samples were macro dissected, selecting the most preserved areas of HCC. The area of interest was cut at 10 micra thick slices, which were deparaffinized using Xylene and Ethanol. Paraffin free samples were digested with a 20 mg/ml Proteinase K solution and lysis buffer. DNA was extracted by DNeasy^® ^Tissue Mini-Kit (Qiagen, Hilden, Germany), according to the manufacturer's instructions and eluted in 150 μl of Elution Buffer. The concentration and the integrity of the DNA were analyzed by 1.5% agarose gel electrophoresis, using Low Mass DNA Ladder (Invitrogen, Carlsbad, California) as marker.

### DNA Amplification

The selected primers for this study flank TP53 exon 7 and were designed according to Lehman [24] in order to amplify the whole sequence of the target exon. Five μl of the eluted DNA was used as template for the PCR at a final concentration of 100 nM of each primer (forward and reverse); 0.1 mM of each dNTP; 1.5 mM of MgCl_2_; 1× reaction Buffer and MilliQ water completing the reaction to a final volume of 50 μl. No-template-controls were used for each PCR set to check for contamination.

PCR products were quantified using Low Mass Ladder (Invitrogen Carlsbad, California).

For DNA amplification with mutant specific primer, an additional reverse primer (5'tgt gat gat ggt gag gat ggg a 3') was added to the PCR reaction described above. A wild type TP 53 control was used as negative control for TP-53 mutation.

### Mutation Analysis

#### Restriction Fragment Length Polymorphism (RFLP)

Specific G to T transversion at codon 249 of exon 7 of TP53 was analyzed through RFLP. One unit of *HaeIII *(a 10 fold excess of enzyme) was added in 100 ng of p53 PCR product with its specific reaction buffer. The reaction was incubated at 37°C for 4 hours. Afterwards, an electrophoresis of the restricted PCR product in a 3% agarose 1,000 gel (Invitrogen, Carlsbad, USA) was performed. A 158 bp DNA fragment denoted the specific mutation.

#### Sequencing

In order to validate the results from RFLP, exon 7 region of the TP53 gene was directly sequenced. 30 ng of PCR product was used as template for sequencing reaction; 3.2 picomols of primer and 6 μl of Big Dye Terminator (Applied Biosystems, Foster City, USA), the reaction volume was completed with MilliQ water to a final volume of 20 μl. It was used ABI 377 automated sequencer (AppliedBiosystem, Foster City, USA).

### Assessment of the presence of HBV DNA in liver tissue

A qualitative assay using Real-Time PCR was performed to detect HBV DNA in the eluted total DNA from the hepatic tissue from all cases and controls, with primers and probes from the Protein S region of the HBV genome. Both primers and probes were designed to cover all A, B, C, D, E and F HBV genotypes (primers have not been published yet). Briefly 5 μl of the DNA solution was used as template along with 1× TaqMan mastermix (Roche), 100 nM of each primer and 50 nM of a 5'FAN 3'MGB probe. It was used the 7300 Real-Time thermocycler (Applied Biosystems, Foster City, USA).

### Statistical analysis

Univariate statistical analysis was performed using *x*^2 ^test and Student's *t*-test. Upon completion of the univariate analyses, variables were select for the multivariable analysis. Any variable whose univariate test had a p-value < 0.25 was considered as a candidate for multivariate model along with all variables of known biologic importance. The 0.25 level was chosen as a screening criterion because studies have shown that using a lower level (e.g. the traditional 0.05 level) often fails to identify variables known to be important. And the populated multivariate model will control for bias and exclude those with the p level of 0.05. Therefore following the above criteria the following variables were chosen for multivariate analysis: age, sex, ethnic origin, presence of cirrhosis, tumor differentiation grade, tumor size, vascular invasion and HBV positivity by Real Time PCR. The multivariate logistic regression used a forward selection procedure and only those variables with significant p at 0.05 were included in the final model. All p values reported are for a two-sided test, and the level of significance was set at 0.05.

## Results

### Pathological data

From the 74 FFPE blocks in which yielded DNA amplification, 49 (66.2%) presented liver cirrhosis. The mean tumor size was 9.08 cm (0.5 – 24 cm). Grossly the tumor showed vascular invasion in (45.9%) of the samples.

Tumor grade was classified according to the Edmond-Steiner criteria, in which G1 is a well-differentiated tumor and G4 is the most undifferentiated form. Of all samples 1/74 (1.35%) case was classified G1, 30/74 (40.5%) G2, 34/74 (45.9%) G3 and 9/74 (12.2%) G4.

### Clinical Data

Fifty-three were male patients (71.6%) and the average was 54.04 years. The HCV infection data was obtained from Clinical Hospital of Sao Paulo University School of Medicine electronic system and from CICAP – German Hospital Oswaldo Cruz in 56 patients whereas HBV DNA by Real-Time PCR was achieved in all 74 patients. The resulting "viral status" showed that 27/56(48.2%) were HCV infected, 13/74 (17.6%) HBV infected, 3/56 (5.4%) co-infected and 12/56(21.4%) with no viral infection.

### DNA Extraction and PCR Amplification

DNA extraction was performed as described above. The mean DNA concentration extracted was 40 ng/ml. Even though the extraction was successfully done in all samples, 6 of them was not observed PCR amplification and were excluded from the study. The absence of amplification in those samples may be due to an excessive degradation of the genomic DNA.

### Mutation and HBV DNA Presence

**Figure 1:**
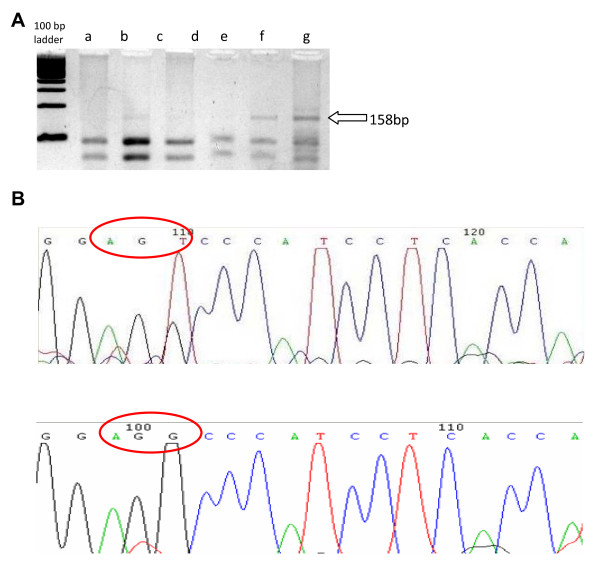
**A. RFLPs of PCR-amplified fragments obtained with *HaeIII***. There are 2 genotypes represented by the gel picture, wild type (a, c and d) where a 158 bp band is absent and 249^Ser ^(b, e and f) where the 158 pb fragment can be observed. 1B. Representative eletropherograms of TP53 sequence. Nucleotide sequence of a mutated sample on top, showing the G → T transversion (AGT) when compared with a wild type exon 7 below (AGG).

**Figure 2:**
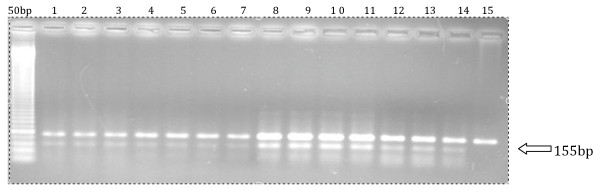
**PCR-amplified products with mutant-specific primer for identification of mutant TP-53 allele (lower 155 bp band)**. Lane 15 is a wild type control.

249^Ser ^mutation was found in 21/74 samples (28%) by RFLP and all samples were also submitted to sequencing (Figure 1). The presence of mutation was confirmed in 12 of 21 samples by nucleic acid sequencing (Figure 1). No mutation was detected in any of the 17 controls. To further demonstrate the presence of the TP-53 mutant form, an alternative PCR amplification with a reverse TP-53 mutant-specific primer was performed in 14 of the 21 samples available for PCR. A 155 band confirmed the presence of the mutant form. A wild type TP 53 DNA (Lane 15) was used as a wild form control (Figure 2). In all 14 samples, the intensity of mutant p53-specific band was lower than that of wild-type p53, suggesting that all tumors retained wild-type p53 or alternatively a fraction of tumor cells displayed the p53-249ser mutation.

The assessment of sensibility of Real-Time qualitative assay was done by a serial dilution of pSM2 – a plasmid containing the whole genome of HBV described by Günther [25]. The minimum amount of HBV DNA necessary for detection was 5 copies per reaction.

Using the same parameters from the standardization, it was observed HBV DNA amplification in 13/74 (16%) samples

### Variables related to 249^Ser^

The mutation frequency according to HCC differentiation level is described in table 2. G4 tumors had a tendency to have higher frequency of 249^Ser ^when compared to the other levels (p = 0.054, NS).

**Table 2:** Mutation distribution according to Edmondson & Steiner histological grade

	Histological Grade				
	
	1	2	3	4	Total
**Wild-Type 249**	1	23	26	3	**53**
**249^Ser^**	0	7	8	6	**21**

**Total**	**1**	**30**	**3**	**9**	**74**

p = 0.054

Tumor size data was available for 68 samples. Mean tumor samples size presenting 249^Ser ^was 9.4 cm, significantly larger (p = 0.015) than that found in cases without this mutation, which was 5.5 cm as described on table 3.

**Table 3:** Size difference between tumors according to 249^Ser ^mutation

Codon 249	Samples	Mean Size (cm)
**Wild-Type 249**	49*	5.5 ± 4.9
**249^Ser^**	19*	9.4 ± 7.4

p = 0.015

* Size information available for 68 samples

As it can be seen on table 4, histological grade, and tumor size increases the odds of presenting 249^Ser^: OR = 2.415(1.001 – 5.824) and 1.10 (1.001 – 1.214) respectively. Male gender had a borderline result of OR = 4.9173 (0.954 – 25.345).

**Table 4:** Multivariable analysis of 249^Ser ^associated factors

	Odds Ratio	95% CI	p
**Grade**	2.415	1.001 – 5.824	0.050
**Male Gender**	4.917	0.954 – 25.345	0.057
**Tumor Size**	1.102	1.001 – 1.214	0.047

Mean patient age with or without 249^Ser ^was not significantly different (53.05 ± 12.35 years versus 54.41 ± 18.98 years, p = 0.3825). Mutation frequency tended to be higher in HCC from cirrhotic liver (34.7%) than non-cirrhotic (16%). However this difference was not significant (p = 0.076, NS). Mutation frequency was 3/21 (14.28%) in female and 18/53 (33.9%) in male (p = 0.076, NS).

HBV presence had a 1.150 (0.312 – 4.237) OR (p = 0.53768) suggesting no relationship between HBV DNA presence and 249^Ser^. Neither 249^Ser ^nor HBV DNA presences were related to vascular invasion (p = 0.470): OR = 0.84 (0.303 – 2.326) and p = 0.611 OR = 1.010 (0.303 – 3.357).

No additional mutation was detected in any other hotspot of TP53 exon 7 codon 249.

## Discussion

The 249^Ser ^mutation was found in 28% of HCC samples included in this study, it is lower somewhat similar to our previous finding of immunohistochemistry reactivity for p53 in 35% of Brazilian HCC cases from our group [26]. At that time only immunoexpression of this protein was analyzed, not the determination of the mutation at the hotspot(s) responsible for this over expression.

The 28% rate of 249^Ser ^TP53 point mutation found is rather high, a finding probably related to previous studies of contamination of Brazilian food with aflatoxin [27,1]. AFB1 contamination is a public health problem in Brazil. It is also important to remind that codon 249 is the responsible for 33% of TP53 mutations in HCC, but it is not the only TP53 hotspot. In a search on IARC database, it is possible to find other TP53 hotspots. It is described other 143 mutations besides 249^Ser ^in HCC. Other common mutation takes place in codons 175, 245, 248 273, and 282. The other codons have an individual contribution to TP53 mutation of less than 2% [28]. This information can justify the difference between the frequency of the mutation found in this study and the higher frequency of over expressed p53 described previously [26].

Our results are strikingly differing to those published in European countries where, not only 249^Ser^, but also all TP53 hotspots do not have relevant influence in HCC carcinogenesis. Kubicka [29] showed that none of his 20 HCC samples had the 249^Ser ^point mutation and between all cases only one had p53 over expression due to a 248 codon mutation. USA had a similar result: even though there was 5/23 cases of HCC with p53 over expression all of them were wild type for the 249 codon [30].

It was described a 45% frequency of p53 over expression on his samples – a high rate for Europeans samples – even though no codon 249 mutation was found [31].

On the other hand, in countries with high incidence of HCC, the 249^Ser ^point mutation has an important role in the liver carcinogenesis. In Senegal this kind of mutation can be detected in 67% of HCC patient [32]. In regions like Gambia (Africa) and Qidong (China) this mutation frequency can reach up to 50%. The mutation rate found in our study is much higher comparing to European countries yet lower than in regions where aflatoxin exposure is endemic. Unfortunately, there are not many studies about 249^Ser ^frequency in Latin-American countries. However, in Mexico it was described a 3/16 (19%) frequency of this mutation in HCC [33]. This 249^Ser ^frequency may be related to the fact that Mexico is one of the biggest corn consumers in the world (almost 120 kg per capita per year). This fact associated with an imperfect foodstuff storage conditions and manipulation may result in a large AFB1 intake. Another fact which should be discussed is the fact that up to 2005, Mexican legislation about Aflatoxin contamination lack of rules about Aflatoxin presence in milk [34].

Although, AFB1 contamination has been controlled in Brazil, there are still cases of foods presenting AFB1 levels beyond the tolerated level, for example: 20 kinds of peanuts were interdicted during February 2005 and July 2006 by ANVISA (National Sanitary Administration Agency) [35]. The maximum AFB1 level in food allowed by ANVISA was 30 ppb up to 2002 and 20 ppb after that. FDA has always recommended an AFB1 level less than 15 ppb. European Food Safety Authority (EFSA) allows only 0.1 ppb of AFB1 in some cases and there are specific rules about food for child nutrition [36]. Moreover, residual effects of exposition to higher aflatoxin levels before 2002 will probably yield high rates of AFB-related HCC in Brazil for the next few decades.

Our data might suggest that 249^Ser ^is related not only to poorly differentiated HCC but also to also larger tumors. It was showed that mutations among TP53 are associated with poor differentiation level [37,38]. However, in both studies there was a low 249^Ser ^frequency and the poor differentiation was associated only to TP53 mutation and not to 249^Ser ^itself. Moreover, neither of those studies found association between TP53 mutation and tumor size. Age and vascular invasion did not present correlation with the specific mutation. Despite not presenting statistical relationship with 249^Ser^, the mutation had a tendency of being more frequent in cirrhotic liver. The same can be said about gender: The mutation was observed slightly more frequently in males rather in female ones (p = 0.076).

The fact that the mutation was more frequent among larger and less differentiated tumors could suggest that 249^Ser ^is a late event on liver carcinogenesis, which may sound, be against the hypothesis that AFB1 is a causal agent of HCC. However, there are studies that describe the selective advantages that a liver cell carrying this mutation may have. Among them it could be pointed: enhancement of cell growth [39], inhibition of wild-type p53 mediated apoptosis [40] and finally p53 249^ser ^has great efficiency in suppressing wild-type p53 activity [41].

Interestingly, all HCC samples had low levels of mutant p53-249 ser mutation as compared to wild-type p53 DNA. Because larger and less differentiated tumors are less homogeneous it is possible that only part of tumor displayed the p53-249^ser ^mutation, following the suggesting that 249^ser ^is a late event on liver carcinogenesis. Alteration in p53 may be important event in the transformation of hepatocytes of regenerative nodules in a damaged organ (the "field effect") to the malignant phenotype. Also it is possible that part of non-tumor tissue with wild-type p53DNA contributed for this situation, since it was not performed micro dissection to separated only tumor cells. Nevertheless, this situation did not preclude the detection p53 249^ser ^mutation in a significative number of tumors, suggesting a role of aflatoxin in the carcinogenesis of liver tumor in our country.

Statistical relationship between p53 249^ser ^mutation and HBV presence in the hepatocytes was not found. This, however, may be due to the fact that only HBV DNA presence was analyzed and not its gene expression. Many studies describe that the pathway, which leads to the 249^ser ^mutation, is related with HBx expression however it was not this project the aim. In this study HBV DNA presence was not found being related to neither cirrhosis status nor vascular invasion of the tumor.

## Conclusion

In conclusion, the frequency of 249^Ser ^found in this study was 28%, which may suggest AFB1 exposure and indicating that this mutation is an important factor of HCC carcinogenesis in Brazil. HBV DNA presence did not show to be a hazard factor to 249^Ser ^development. However, it was observed a relationship between poorly differentiated HCC and tumor size to this specific mutation.

Even though the mutation frequency found in this study was higher than those found in low HCC incidence areas, it was still lower than countries with moderate AFB1 exposure like Mexico.

## Competing interests

The authors declare that they have no competing interests.

## Authors' contributions

JAN carried out the collection of the data, performed the laboratory experiments, the statistical analysis with interpretation, and drafted the manuscript. SKON contributed with the conception and design of the study, revised, acted as corresponding author and approved the final manuscript. MEN contributed with the conception, design of the study, carried out the collection of the data, the statistical analysis with interpretation, and revised the manuscript. MMTS and EPC contributed doing the laboratory experiments. DVP carried out the collection of the data. ESM, CS and VAFA supplied the samples, carried out the pathological staging, and revised the manuscript. FJC contributed with the conception and design of the study, revised, and approved the final manuscript. All authors were involved in the research presented and approved the final manuscript.

## Pre-publication history

The pre-publication history for this paper can be accessed here:

http://www.biomedcentral.com/1471-2407/9/204/prepub

## References

[B1] Strosnider H Azziz-Baumgartner E Banziger M Bhat RV Breiman R Brune MN DeCock K Dilley A Groopman J Hell K Workgroup report: public health strategies for reducing aflatoxin exposure in developing countries Environmental health perspectives 2006 114 12 1898 1903 17185282 10.1289/ehp.9302 PMC1764136

[B2] Thorgeirsson SS Grisham JW Molecular pathogenesis of human hepatocellular carcinoma Nature genetics 2002 31 4 339 346 10.1038/ng0802-339 12149612

[B3] Nita ME Alves VA Carrilho FJ Ono-Nita SK Mello ES Gama-Rodrigues JJ Molecular aspects of hepatic carcinogenesis Revista do Instituto de Medicina Tropical de Sao Paulo 2002 44 1 39 48 11896411 10.1590/s0036-46652002000100007

[B4] Rocken C Carl-McGrath S Pathology and pathogenesis of hepatocellular carcinoma Digestive diseases (Basel, Switzerland) 2001 19 4 269 278 11935086 10.1159/000050693

[B5] El-Serag HB Rudolph KL Hepatocellular carcinoma: epidemiology and molecular carcinogenesis Gastroenterology 2007 132 7 2557 2576 10.1053/j.gastro.2007.04.061 17570226

[B6] Bosch FX Ribes J Borras J Epidemiology of primary liver cancer Seminars in liver disease 1999 19 3 271 285 10.1055/s-2007-1007117 10518307

[B7] El-Serag HB Davila JA Petersen NJ McGlynn KA The continuing increase in the incidence of hepatocellular carcinoma in the United States: an update Annals of internal medicine 2003 139 10 817 823 14623619 10.7326/0003-4819-139-10-200311180-00009

[B8] Bressac B Kew M Wands J Ozturk M Selective G to T mutations of p53 gene in hepatocellular carcinoma from southern Africa Nature 1991 350 6317 429 431 10.1038/350429a0 1672732

[B9] Hsu IC Metcalf RA Sun T Welsh JA Wang NJ Harris CC Mutational hotspot in the p53 gene in human hepatocellular carcinomas Nature 1991 350 6317 427 428 10.1038/350427a0 1849234

[B10] Ozturk M p53 mutation in hepatocellular carcinoma after aflatoxin exposure Lancet 1991 338 8779 1356 1359 10.1016/0140-6736(91)92236-U 1682737

[B11] Jackson PE Kuang SY Wang JB Strickland PT Munoz A Kensler TW Qian GS Groopman JD Prospective detection of codon 249 mutations in plasma of hepatocellular carcinoma patients Carcinogenesis 2003 24 10 1657 1663 10.1093/carcin/bgg101 12869416

[B12] Kirk GD Camus-Randon AM Mendy M Goedert JJ Merle P Trepo C Brechot C Hainaut P Montesano R Ser-249 p53 mutations in plasma DNA of patients with hepatocellular carcinoma from The Gambia Journal of the National Cancer Institute 2000 92 2 148 153 10.1093/jnci/92.2.148 10639517

[B13] Tiemersma EW Omer RE Bunschoten A van't Veer P Kok FJ Idris MO Kadaru AM Fedail SS Kampman E Role of genetic polymorphism of glutathione-S-transferase T1 and microsomal epoxide hydrolase in aflatoxin-associated hepatocellular carcinoma Cancer Epidemiol Biomarkers Prev 2001 10 7 785 791 11440964

[B14] Smela ME Currier SS Bailey EA Essigmann JM The chemistry and biology of aflatoxin B(1): from mutational spectrometry to carcinogenesis Carcinogenesis 2001 22 4 535 545 10.1093/carcin/22.4.535 11285186

[B15] Puisieux A Lim S Groopman J Ozturk M Selective targeting of p53 gene mutational hotspots in human cancers by etiologically defined carcinogens Cancer research 1991 51 22 6185 6189 1933877

[B16] Denissenko MF Cahill J Koudriakova TB Gerber N Pfeifer GP Quantitation and mapping of aflatoxin B1-induced DNA damage in genomic DNA using aflatoxin B1–8,9-epoxide and microsomal activation systems Mutation research 1999 425 2 205 211 10216213 10.1016/s0027-5107(99)00038-x

[B17] Guengerich FP Johnson WW Shimada T Ueng YF Yamazaki H Langouet S Activation and detoxication of aflatoxin B1 Mutation research 1998 402 1–2 121 128 9675258 10.1016/s0027-5107(97)00289-3

[B18] Su Q Schroder CH Hofmann WJ Otto G Pichlmayr R Bannasch P Expression of hepatitis B virus X protein in HBV-infected human livers and hepatocellular carcinomas Hepatology (Baltimore, Md) 1998 27 4 1109 1120 10.1002/hep.510270428 9537452

[B19] Kim CM Koike K Saito I Miyamura T Jay G HBx gene of hepatitis B virus induces liver cancer in transgenic mice Nature 1991 351 6324 317 320 10.1038/351317a0 2034275

[B20] Su F Schneider RJ Hepatitis B virus HBx protein activates transcription factor NF-kappaB by acting on multiple cytoplasmic inhibitors of rel-related proteins Journal of virology 1996 70 7 4558 4566 8676482 10.1128/jvi.70.7.4558-4566.1996 PMC190392

[B21] Hosono S Chou MJ Lee CS Shih C Infrequent mutation of p53 gene in hepatitis B virus positive primary hepatocellular carcinomas Oncogene 1993 8 2 491 496 8093978

[B22] Qu JH Zhu MH Lin J Ni CR Li FM Zhu Z Yu GZ Effects of hepatitis B virus on p53 expression in hepatoma cell line SMMU-7721 World J Gastroenterol 2005 11 39 6212 6215 16273653 10.3748/wjg.v11.i39.6212 PMC4436643

[B23] Lunn RM Zhang YJ Wang LY Chen CJ Lee PH Lee CS Tsai WY Santella RM p53 mutations, chronic hepatitis B virus infection, and aflatoxin exposure in hepatocellular carcinoma in Taiwan Cancer research 1997 57 16 3471 3477 9270015

[B24] Lehman TA Bennett WP Metcalf RA Welsh JA Ecker J Modali RV Ullrich S Romano JW Appella E Testa JR p53 mutations, ras mutations, and p53-heat shock 70 protein complexes in human lung carcinoma cell lines Cancer research 1991 51 15 4090 4096 1855224

[B25] Gunther S Li BC Miska S Kruger DH Meisel H Will H A novel method for efficient amplification of whole hepatitis B virus genomes permits rapid functional analysis and reveals deletion mutants in immunosuppressed patients Journal of virology 1995 69 9 5437 5444 7636989 10.1128/jvi.69.9.5437-5444.1995 PMC189390

[B26] Alves VA Nita ME Carrilho FJ Ono-Nita SK Wakamatsu A Lehrbach DM de Carvalho MF de Mello ES Gayotto LC da Silva LC p53 immunostaining pattern in Brazilian patients with hepatocellular carcinoma Revista do Instituto de Medicina Tropical de Sao Paulo 2004 46 1 25 31 15057330 10.1590/s0036-46652004000100005

[B27] Sabino M Prado G Inomata EI Pedroso Mde O Garcia RV Natural occurrence of aflatoxins and zearalenone in maize in Brazil. Part II Food additives and contaminants 1989 6 3 327 331 10.1080/02652038909373787 2524411

[B28] Robins H Alexa G Harris S Levine AJ Hainaut P, Wiman KG The first twenty-five years of p53 research 25 years of p53 research 2007 The Netherlands Springer 1 25

[B29] Kubicka S Trautwein C Schrem H Tillmann H Manns M Low incidence of p53 mutations in European hepatocellular carcinomas with heterogeneous mutation as a rare event Journal of hepatology 1995 23 4 412 419 10.1016/0168-8278(95)80199-5 8655958

[B30] Hoque A Patt YZ Yoffe B Groopman JD Greenblatt MS Zhang YJ Santella RM Does aflatoxin B1 play a role in the etiology of hepatocellular carcinoma in the United States? Nutrition and cancer 1999 35 1 27 33 10.1207/S1532791427-33 10624703

[B31] Volkmann M Hofmann WJ Muller M Rath U Otto G Zentgraf H Galle PR p53 overexpression is frequent in European hepatocellular carcinoma and largely independent of the codon 249 hot spot mutation Oncogene 1994 9 1 195 204 8302580

[B32] Coursaget P Depril N Chabaud M Nandi R Mayelo V LeCann P Yvonnet B High prevalence of mutations at codon 249 of the p53 gene in hepatocellular carcinomas from Senegal British journal of cancer 1993 67 6 1395 1397 8390289 10.1038/bjc.1993.258 PMC1968506

[B33] Soini Y Chia SC Bennett WP Groopman JD Wang JS DeBenedetti VM Cawley H Welsh JA Hansen C Bergasa NV An aflatoxin-associated mutational hotspot at codon 249 in the p53 tumor suppressor gene occurs in hepatocellular carcinomas from Mexico Carcinogenesis 1996 17 5 1007 1012 10.1093/carcin/17.5.1007 8640905

[B34] Garcia S Heredia N Mycotoxins in Mexico: epidemiology, management, and control strategies Mycopathologia 2006 162 3 255 264 10.1007/s11046-006-0058-1 16944292

[B35] ANVISA http://www.anvisa.gov.br/inspecao/alimentos/interditados_2006.htm

[B36] Byrne D Amending Regulation (EC) No 466/2001 as regards aflatoxins and ochratoxin A in foods for infants and young children Official Journal of the European Union. COMMISSION REGULATION (EC) No 683/2004 of 13 April 2004. L106/3 – 5 http://eur-lex.europa.eu/LexUriServ/site/en/oj/2004/l_106/l_10620040415en00030005.pdf

[B37] Oda T Tsuda H Scarpa A Sakamoto M Hirohashi S p53 gene mutation spectrum in hepatocellular carcinoma Cancer research 1992 52 22 6358 6364 1330291

[B38] Ng IO Chung LP Tsang SW Lam CL Lai EC Fan ST Ng M p53 gene mutation spectrum in hepatocellular carcinomas in Hong Kong Chinese Oncogene 1994 9 3 985 990 8108145

[B39] Ponchel F Puisieux A Tabone E Michot JP Froschl G Morel AP Frebourg T Fontaniere B Oberhammer F Ozturk M Hepatocarcinoma-specific mutant p53-249ser induces mitotic activity but has no effect on transforming growth factor beta 1-mediated apoptosis Cancer research 1994 54 8 2064 2068 8174105

[B40] Wang XW Gibson MK Vermeulen W Yeh H Forrester K Sturzbecher HW Hoeijmakers JH Harris CC Abrogation of p53-induced apoptosis by the hepatitis B virus X gene Cancer research 1995 55 24 6012 6016 8521383

[B41] Forrester K Lupold SE Ott VL Chay CH Band V Wang XW Harris CC Effects of p53 mutants on wild-type p53-mediated transactivation are cell type dependent Oncogene 1995 10 11 2103 2111 7784055

